# Are Inflammatory Biomarkers at ICU Discharge Still Predictive of Post-ICU Mortality in Sepsis and Septic Shock? A Retrospective, Single-Center Cohort Study

**DOI:** 10.3390/jcm15114111

**Published:** 2026-05-26

**Authors:** Mustafa Ay, Rabia Sari

**Affiliations:** 1 Intensive Care Unit, Antalya Training and Research Hospital, 07100 Antalya, Türkiye; 2 Intensive Care Unit, Okmeydani Training and Research Hospital, 34384 Istanbul, Türkiye; drrabiasari@yahoo.com

**Keywords:** sepsis, septic shock, inflammatory biomarkers, neutrophil-to-lymphocyte ratio, ICU discharge, post-ICU mortality, C-reactive protein-to-albumin ratio, risk stratification

## Abstract

**Background:** Sepsis and septic shock are associated with high mortality in intensive care units (ICUs), with a substantial risk persisting after ICU discharge. However, it remains unclear whether inflammatory biomarkers retain their prognostic value at the time of ICU discharge. This study aimed to evaluate whether discharge inflammatory biomarkers—including neutrophil-to-lymphocyte ratio (NLR), platelet-to-lymphocyte ratio (PLR), platelet-to-mean platelet volume ratio (PLT/MPV), and C-reactive protein-to-albumin ratio (CAR)—remain predictive of short- and long-term mortality in patients with sepsis and septic shock. **Methods:** In this single-center, retrospective cohort study, adult patients with sepsis or septic shock discharged from a tertiary ICU specializing in chest diseases between January 2013 and January 2015 were included. Sepsis and septic shock were retrospectively re-classified according to Sepsis-3 criteria. Inflammatory biomarkers measured at ICU admission and discharge, along with clinical variables and disease severity scores (APACHE II and SOFA), were recorded. Patients were followed for 28-day, 6-month, and 2-year mortality. The prognostic performance of biomarkers was assessed using receiver operating characteristic (ROC) analysis, and optimal cut-off values were determined. Independent predictors of mortality were evaluated using Cox proportional hazards regression analysis. **Results:** A total of 461 patients were included. In total, 291 (63.1%) had sepsis without shock and 170 (36.9%) had septic shock. The overall male proportion was 62%, with a median age of 65 (IQR 54–74) years in the sepsis group and 70 (63–79) years in the septic shock group. Mortality rates were significantly higher in patients with septic shock compared to those with sepsis at 28 days (24% vs. 10%, *p* < 0.001), 6 months (44% vs. 27%, *p* < 0.001), and 2 years (71% vs. 57%, *p* = 0.003). In unadjusted survivor/non-survivor comparisons, elevated discharge NLR and CAR were associated with early post-ICU mortality. However, in multivariable Cox regression, discharge NLR, but not discharge CAR, remained independently associated with 28-day and 6-month mortality. On ROC analysis, discharge NLR showed moderate discriminative performance for 28-day mortality (AUC 0.67, 95% CI 0.60–0.74), as did discharge CAR (AUC 0.68, 95% CI 0.60–0.76), although CAR did not retain independent prognostic significance after adjustment. An NLR value ≥ 5 was identified as an independent predictor of 28-day mortality (HR 2.44; 95% CI 1.24–4.80; *p* = 0.010) and was also significantly associated with 6-month mortality (HR 2.02; 95% CI 1.18–3.45; *p* = 0.011), although its predictive value decreased over longer follow-up periods (HR 1.37; 95% CI 0.93–2.01; *p* = 0.11 at 2 years). **Conclusions:** Inflammatory biomarkers measured at ICU discharge, particularly NLR, remain predictive of short-term mortality in patients with sepsis and septic shock, but their prognostic value diminishes over time. Assessment of inflammatory status at ICU discharge may provide a practical tool for early post-ICU risk stratification and may support clinical decisions regarding intensified outpatient surveillance and follow-up scheduling in this vulnerable population.

## 1. Introduction

Sepsis is one of the leading causes of mortality and morbidity in intensive care units (ICUs) and represents a major global public health problem. Global epidemiological data indicate that millions of patients are hospitalized with sepsis each year, with a substantial proportion developing organ failure. Despite advances in critical care, mortality rates remain high, particularly among patients who develop septic shock, where reported mortality ranges between 30% and 50% [1,2,3]. In clinical practice, disease severity scoring systems such as the Acute Physiology and Chronic Health Evaluation II (APACHE II) and the Sequential Organ Failure Assessment (SOFA) are widely used to predict prognosis in patients with sepsis [4,5]. In addition, inflammatory biomarkers such as C-reactive protein (CRP) are frequently used to assess disease severity and predict clinical outcomes [6].

In recent years, increasing attention has been directed toward inflammatory biomarkers derived from routine laboratory parameters for predicting the prognosis of sepsis. The neutrophil-to-lymphocyte ratio (NLR), reflecting the balance between neutrophil-mediated inflammation and lymphocyte-mediated adaptive immune response, has been associated with mortality in patients with sepsis [7,8]. The C-reactive protein-to-albumin ratio (CAR), which reflects both systemic inflammation and nutritional status, has also been linked to disease severity and mortality [9,10]. Similarly, platelet-related indices such as the platelet-to-lymphocyte ratio (PLR) and platelet-to-mean platelet volume ratio (PLT/MPV) have been shown to correlate with inflammatory response and disease severity [11,12].

However, most studies to date have focused on biomarker levels measured at ICU admission and their association with in-hospital mortality. Emerging evidence suggests that persistent inflammation, immune dysfunction, and metabolic alterations following sepsis may significantly influence long-term outcomes. This constellation of post-sepsis pathophysiology has been conceptualized as Persistent Inflammation, Immunosuppression, and Catabolism Syndrome (PICS), which contributes to chronic critical illness, recurrent infections, and increased late mortality [13,14,15]. Furthermore, mortality risk has been shown to persist well beyond ICU discharge in patients with sepsis [16,17]. In this context, it remains unclear whether inflammatory biomarkers retain their prognostic value at the time of ICU discharge and continue to predict outcomes in the post-ICU period.

Therefore, the present study aimed to evaluate whether inflammatory biomarkers measured at ICU discharge remain predictive of short- and long-term mortality in patients with sepsis and septic shock. We hypothesized that inflammatory biomarkers measured at ICU discharge would retain prognostic value for short-term mortality, but that their predictive performance would attenuate over longer follow-up periods, reflecting a transition from acute inflammation-driven to comorbidity-driven mortality.

## 2. Materials and Methods

This single-center retrospective observational cohort study was conducted in a level III intensive care unit (ICU) of a university hospital specializing in chest diseases and thoracic surgery between January 2013 and January 2015. Ethical approval was obtained from the local ethics committee (approval number: 116.2017.006, dated 25 May 2017). Due to the retrospective design, the requirement for informed consent was waived. The study was conducted in accordance with the Declaration of Helsinki and reported following the Strengthening the Reporting of Observational Studies in Epidemiology (STROBE) guidelines. At the time of patient enrollment, the Sepsis-2 criteria were in clinical use; following publication of the Sepsis-3 consensus definitions in 2016, all cases were retrospectively re-classified according to the Sepsis-3 criteria, and only patients fulfilling these definitions were included in the final analysis.

Patient selection was based on the following criteria:
Inclusion criteria:

- Adult patients aged ≥ 18 years;
- Diagnosis of sepsis or septic shock according to the Sepsis-3 criteria;
- Admission to the ICU;
- Survival to both ICU and hospital discharge.

Exclusion criteria:

- ICU mortality;
- Post-ICU in-hospital mortality;
- Advanced malignancy or any active oncologic condition;
- Active rheumatologic disease;
- Age < 18 years;
- Missing or incomplete data.

Eligible patients were followed for up to two years after ICU discharge to assess post-ICU mortality.

**Figure 1:**
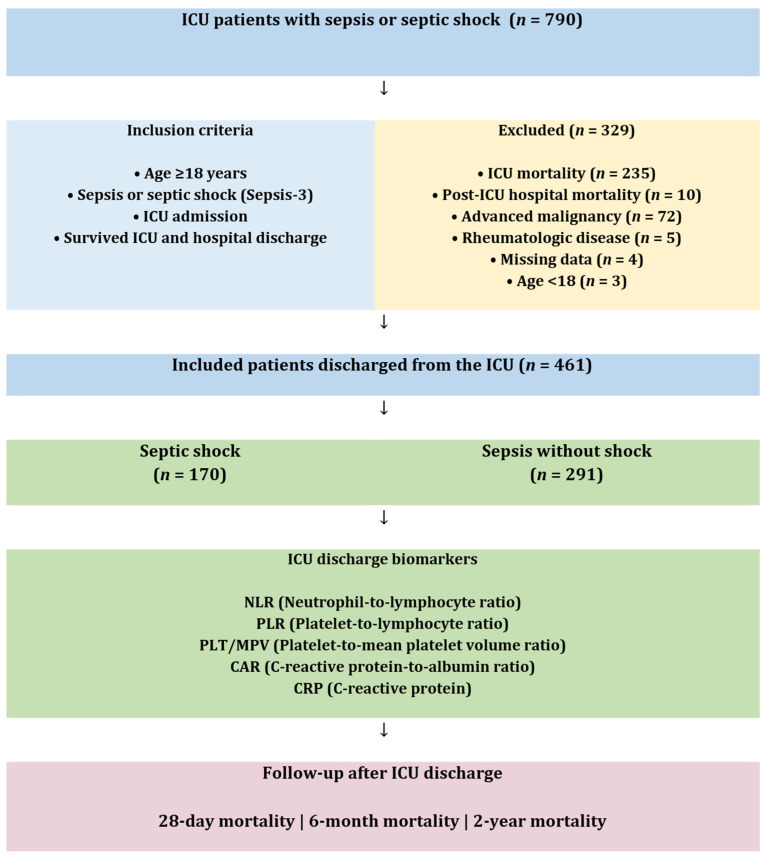
Flow diagram of patient selection and follow-up.

A total of 790 patient records were retrospectively reviewed. Of these, 235 patients who died in the ICU, 10 patients who developed in-hospital mortality after ICU discharge, 72 patients with advanced-stage malignancy, 5 patients with rheumatologic diseases, 4 patients with missing data, and 3 patients aged under 18 years were excluded from the study. Data from a total of 461 patients were analyzed, and these patients were longitudinally followed for two years after ICU discharge to determine early and long-term mortality. Among the included patients, 36.9% (*n* = 170) had septic shock, while 63.1% (*n* = 291) had sepsis without shock (Figure 1).

The primary objective of this study was to evaluate whether inflammatory biomarkers measured at ICU discharge remain predictive of post-ICU mortality. The primary endpoint was 28-day mortality following ICU discharge. Secondary endpoints included 6-month and 2-year mortality, representing the subacute and long-term phases of post-ICU mortality, respectively. One-year mortality is reported descriptively in Table 1 to enable comparison with previous post-sepsis cohort studies; however, it was not pre-specified as a separate analytic endpoint for detailed biomarker performance analyses (ROC and Cox), as it was considered to provide largely overlapping information with the 6-month subacute phase. All 461 patients were followed for a minimum of 24 months after ICU discharge, with mortality status assessed through the national electronic death notification system.

A formal a priori sample size calculation was not performed, as this study included all consecutive eligible patients identified during the pre-defined enrollment period. The adequacy of the available sample for the planned multivariable analyses was nonetheless evaluated using the events-per-variable (EPV) principle for Cox proportional hazards regression, which recommends a minimum of 10 events per candidate covariate to ensure model stability. With 69 events for 28-day mortality, 153 events for 6-month mortality, and 285 events for 2-year mortality, and a maximum of six candidate covariates included in the final models, the EPV ratios exceeded this threshold for all endpoints, supporting the statistical validity of the regression analyses.

### 2.1. Data Collection

Demographic data (age, sex, body mass index, comorbidities), type of mechanical ventilation (invasive, non-invasive, and failure of non-invasive ventilation requiring invasive ventilation), disease severity scores at ICU admission (APACHE II and SOFA), length of ICU stay, and inflammatory biomarker levels at ICU admission and discharge were recorded, including neutrophil-to-lymphocyte ratio (NLR), platelet-to-lymphocyte ratio (PLR), platelet-to-mean platelet volume ratio (PLT/MPV), C-reactive protein-to-albumin ratio (CAR), and C-reactive protein (CRP). Each ratio was calculated from concurrently measured laboratory values obtained on the day of ICU admission and on the day of ICU discharge (defined as values within the last 24 h preceding discharge). As serum albumin was not measured systematically in our chest-disease-specialized ICU during the study period, CAR could be calculated only for patients with available albumin data. Accordingly, CAR at ICU admission was available in 341 of 461 patients (74.0%; 120 missing) and at ICU discharge in 286 of 461 patients (62.0%; 175 missing); CRP values were available in 362/461 (78.5%; 99 missing) at admission and 400/461 (86.8%; 61 missing) at discharge. Analyses involving CAR and CRP were performed as complete-case analyses for the respective variable.

Data were obtained from hospital electronic records, ICU follow-up forms, and institutional archives. Sepsis and septic shock were defined according to the Sepsis-3 criteria. ICU length of stay was defined as the time from admission to discharge. Mortality data up to two years after ICU discharge were obtained from the national electronic death notification system.

### 2.2. Patient Grouping

Patients were categorized into two groups based on ICU admission diagnosis: sepsis and septic shock. Subgroup analyses were also performed according to survival status after ICU discharge (survivors vs. non-survivors).

### 2.3. Statistical Analysis

Statistical analyses were performed using SPSS software (version 20.0; SPSS Inc., Chicago, IL, USA). Continuous variables were expressed as mean ± standard deviation (SD) or median (interquartile range, IQR), and categorical variables as frequencies and percentages.

Normality was assessed using the Kolmogorov–Smirnov test. Comparisons were performed using Student’s *t*-test or the Mann–Whitney U test, as appropriate, and the chi-square test for categorical variables. Receiver operating characteristic (ROC) curve analysis was used to determine area under the curve (AUC) values and optimal cut-off points. Kaplan–Meier analysis was used for survival evaluation. Cox proportional hazards regression analysis was performed to identify independent predictors of mortality. Variables included in the model were selected based on ROC-derived cut-off values. A *p*-value < 0.05 was considered statistically significant.

## 3. Results

A total of 461 patients were included in the final analysis, comprising 291 (63.1%) with sepsis without shock and 170 (36.9%) with septic shock (Figure 1).

Patients in the septic shock group were older than those in the sepsis group (*p* < 0.001) and had a higher prevalence of hypertension and coronary artery disease (*p* = 0.010 for both). APACHE II and SOFA scores at ICU admission were also significantly higher in patients with septic shock (*p* < 0.001 and *p* = 0.001, respectively). The need for invasive mechanical ventilation was more frequent in the septic shock group (81% vs. 26%; *p* < 0.001), as was the requirement for invasive ventilation due to noninvasive ventilation failure (36% vs. 8%; *p* < 0.001). Median ICU length of stay was longer in patients with septic shock [9 (7–14) vs. 6 (3–8); *p* < 0.001]. At ICU admission, NLR was higher (*p* = 0.024) and the PLT/MPV ratio was lower (*p* = 0.016) in the septic shock group. At ICU discharge, CRP and CAR levels remained significantly higher (*p* = 0.007 and *p* = 0.005, respectively), while the PLT/MPV ratio remained lower (*p* = 0.010). Early (28-day and 6-month) and long-term (2-year) mortality rates were significantly higher in patients with septic shock compared to those with sepsis (*p* < 0.001 for 28-day and 6-month mortality; *p* = 0.003 for 2-year mortality) (Table 1).

In the overall cohort, non-survivors had significantly higher levels of NLR (*p* < 0.001 and *p* = 0.024), CAR (*p* < 0.001 and *p* < 0.001), and CRP (*p* < 0.001 and *p* < 0.001) at ICU discharge compared with survivors at both follow-up time points (28 days and 6 months). In the septic shock group, non-survivors had significantly higher levels of NLR (*p* < 0.001 and *p* = 0.040), CAR (*p* = 0.005 and *p* < 0.001), and CRP (*p* = 0.003 and *p* < 0.001) at ICU discharge compared with survivors at both follow-up time points (28 days and 6 months). In the sepsis group, non-survivors had significantly higher discharge NLR, CAR, and CRP levels at 28 days (*p* = 0.003, *p* = 0.020, and *p* = 0.021, respectively). At 6 months, discharge CAR remained significantly higher among non-survivors (*p* = 0.010), whereas discharge NLR and CRP did not reach statistical significance (*p* = 0.15 and *p* = 0.10; respectively). The inflammatory biomarker data of survivors and non-survivors during the 28-day and 6-month follow-up after ICU discharge in both sepsis and septic shock groups are summarized in Table 2.

Table 3 presents the cut-off and AUC values of inflammatory biomarkers and disease severity scores for predicting mortality in all patients, including both sepsis and septic shock groups. In the short term (28 days), the cut-off values for NLR at ICU discharge of 5.0 (AUC 0.67, 95% CI 0.60–0.74) and for CAR of 16.5 (AUC 0.68, 95% CI 0.60–0.76) in patients with sepsis demonstrated moderate discriminative performance for predicting non-survival. For 6-month mortality, the cut-off values for NLR at ICU discharge of 5.0 (AUC 0.59, 95% CI 0.51–0.67) and for CAR of 13 (AUC 0.69, 95% CI 0.61–0.76) also showed moderate discriminative performance. In the long term (two years), the cut-off value for CAR at ICU discharge of 13 (AUC 0.62, 95% CI 0.54–0.69) demonstrated moderate discriminative performance.

Table 4 presents the cut-off and AUC values of inflammatory biomarkers and disease severity scores for predicting mortality in patients with septic shock. In the short term (28 days), a cut-off value of 5 for NLR at ICU discharge (AUC 0.70, 95% CI 0.60–0.79) was found to have the highest AUC value for predicting non-survival. For 6-month mortality, a cut-off value of 5 for NLR at ICU discharge (AUC 0.60, 95% CI 0.51–0.69) also demonstrated a relatively high AUC value. In the long term (two years), no statistically significant difference was detected in inflammatory biomarkers measured at ICU discharge (*p* > 0.05 for all).

Table 5 summarizes the Cox regression models for mortality risk in the overall cohort, including short-term outcomes at 28 days and 6 months, and long-term outcomes at 2 years. An NLR value ≥ 5 at ICU discharge was identified as an independent predictor of early post-ICU mortality, both in the 28-day period (HR 2.44, 95% CI 1.24–4.80) and in the 6-month period (HR 2.02, 95% CI 1.18–3.45). However, no statistically significant difference was observed for long-term mortality at 2 years (*p* > 0.05).

## 4. Discussion

In the present study, we evaluated whether inflammatory biomarkers measured at ICU discharge remain predictive of post-ICU mortality in patients with sepsis and septic shock. Our findings demonstrate that these biomarkers, particularly NLR and CAR, are significantly associated with early mortality following ICU discharge, while their prognostic value diminishes over longer follow-up periods. Mortality was consistently higher in patients with septic shock compared to those with sepsis, both in the early period and during long-term follow-up. These results suggest that inflammatory biomarkers reflect not only the severity of the acute illness but also the persistence of inflammatory and immunological disturbances in the post-ICU phase.

In recent years, inflammatory indices derived from routine laboratory parameters have been increasingly investigated as prognostic tools in sepsis. Among these, the neutrophil-to-lymphocyte ratio (NLR) is a simple and readily available biomarker reflecting the balance between inflammation and immune response. Previous meta-analyses have demonstrated that elevated NLR values are associated with increased mortality risk in septic patients [7,8]. In addition, individual clinical studies have reported that higher NLR thresholds at ICU admission are associated with increased short- and mid-term mortality [18,19]. A key finding of our study is that a relatively lower NLR threshold (≥5) measured at ICU discharge remained significantly associated with early post-ICU mortality. This finding suggests that even modest elevations in NLR at discharge may reflect persistent inflammation and ongoing immune dysregulation. In contrast to higher cut-off values reported at ICU admission, the prognostic relevance of lower NLR levels at discharge highlights its continued predictive value beyond the acute phase of sepsis. These results indicate that NLR may not only reflect disease severity at presentation but also capture residual inflammatory burden during the recovery phase, supporting its role as a practical biomarker for early post-ICU risk stratification.

Another inflammatory index with prognostic relevance in our study was the C-reactive protein-to-albumin ratio (CAR), which reflects both systemic inflammation and the patient’s nutritional and metabolic status. Previous studies have demonstrated that CAR measured at ICU discharge is associated with short-term mortality in patients with sepsis, with moderate discriminative performance [9,10]. Consistent with these findings, elevated CAR levels in our cohort were significantly associated with early post-ICU mortality. However, similar to NLR, the prognostic value of CAR appeared to be more pronounced in the early period and less evident over longer follow-up durations. These findings suggest that CAR may primarily reflect the acute and subacute inflammatory burden rather than long-term risk, supporting its role as a complementary biomarker for early post-ICU risk stratification. It should be noted that the CAR cut-off values identified in our study were derived from our own ROC analysis and have not been externally validated in the sepsis biomarker literature; they should therefore be regarded as exploratory and require confirmation in independent cohorts.

Platelet-related inflammatory indices, including the platelet-to-lymphocyte ratio (PLR) and the platelet-to-mean platelet volume ratio (PLT/MPV), were also evaluated in our study. Previous studies have suggested that these indices may be associated with inflammatory response and mortality in sepsis [12,20]. In addition, other reports have indicated a potential relationship between platelet indices and clinical outcomes in septic patients [21]. However, in our cohort, their prognostic performance was less pronounced compared to NLR and CAR. This discrepancy may be explained by differences in patient characteristics, disease severity, and the dynamic nature of platelet activation during the course of sepsis. These findings suggest that while platelet-related indices may have a role in reflecting inflammatory processes, their ability to remain predictive of post-ICU mortality appears to be limited, particularly when compared with more robust biomarkers such as NLR. Notably, in the septic shock subgroup at 6-month follow-up, PLT/MPV at ICU discharge tended to be lower in non-survivors than in survivors, suggesting a trend toward an inverse association that did not reach statistical significance. This pattern is biologically plausible, as lower PLT/MPV may reflect either relative thrombocytopenia or an elevated mean platelet volume, both of which have been associated with greater inflammatory burden and adverse outcomes in sepsis.

The mechanisms underlying the prognostic value of inflammatory biomarkers in the post-sepsis period are complex and multifactorial. Survivors of sepsis often develop a clinical state characterized by persistent inflammation, immune dysfunction, and ongoing catabolism [13,14,15]. During this phase, continued neutrophil activation, lymphopenia, and impaired adaptive immune responses may contribute to increased vulnerability and adverse outcomes. In this context, elevated inflammatory biomarkers at ICU discharge likely reflect ongoing immune dysregulation rather than solely the severity of the initial insult. This may explain why biomarkers such as NLR and CAR remain predictive of early post-ICU mortality. However, as the influence of acute inflammatory processes diminishes over time and other factors such as comorbidities and long-term complications become more prominent, the prognostic value of these biomarkers appears to decrease. Consistent with previous studies demonstrating sustained post-sepsis mortality risk beyond hospital discharge, Yende et al. reported a one-year mortality rate of approximately 44% in patients with severe sepsis, while a recent Swedish nationwide population-based cohort study confirmed substantially increased long-term mortality and hospital readmission rates among sepsis survivors over up to 12 years of follow-up, with cardiovascular disease, cancer, and recurrent infections being the leading causes of death [16,17]. In line with these findings, our study also demonstrated a progressive increase in mortality over time, particularly among patients with septic shock, a pattern also reported in recent ICU-based sepsis and septic shock cohorts [22]. These results further support the concept that early post-ICU outcomes are strongly influenced by residual inflammatory and immunological disturbances.

From a clinical perspective, our findings suggest several practical applications. The systematic assessment of inflammatory biomarkers—particularly NLR and CAR—at the time of ICU discharge may serve as a simple and widely available tool for early post-ICU risk stratification. Patients with elevated discharge NLR (≥5) or CAR (≥13) may benefit from intensified outpatient surveillance, more frequent follow-up, and proactive screening for recurrent infections and comorbidity exacerbations. Identification of this vulnerable subgroup may also facilitate targeted interventions in future clinical trials aimed at reducing post-ICU mortality.

A particular characteristic of our cohort is the high prevalence of chronic obstructive pulmonary disease (COPD), reflecting the specialty focus of our institution as a tertiary chest diseases and thoracic surgery referral center. Although this might raise concerns regarding generalizability, pulmonary infection represents the most common source of sepsis in mixed ICU populations worldwide, typically accounting for approximately 40–60% of cases [23]. Patients with underlying COPD constitute a particularly vulnerable subgroup with disproportionately high sepsis-related morbidity and long-term mortality. Our cohort therefore provides clinically relevant data for this frequently encountered and high-risk population, while we acknowledge that the prognostic performance of inflammatory biomarkers in non-pulmonary or non-COPD sepsis populations may differ and warrants external validation.

Several directions for future research can be proposed. Multicenter prospective studies including mixed ICU populations are needed to externally validate the prognostic role of NLR and CAR at ICU discharge, particularly in non-pulmonary and non-COPD sepsis cohorts. Future studies could also explore composite biomarker–clinical risk models and evaluate whether structured post-ICU follow-up programs targeting the high-risk subgroup identified at discharge can reduce long-term mortality.

This study has several limitations. First, it was conducted in a single center with a retrospective design, which may limit the generalizability of the findings. Second, inflammatory biomarkers were evaluated at only two time points (ICU admission and discharge), and dynamic changes over time could not be assessed. Third, as in any retrospective observational study, the influence of unmeasured confounders cannot be excluded; variables such as post-discharge rehabilitation intensity, outpatient follow-up adherence, socioeconomic status, recurrent infections, and comorbidity exacerbations were not systematically captured and may have contributed to the attenuation of biomarker prognostic value observed over longer follow-up periods. Fourth, detailed survivor/non-survivor biomarker comparisons in Table 2 were focused on the 28-day and 6-month follow-up periods, where discharge inflammatory biomarkers showed the strongest and most clinically relevant differences. The 2-year prognostic performance was evaluated separately using ROC analyses and Cox regression models, as presented in Table 3, Table 4 and Table 5, and the independent prognostic value of discharge biomarkers appeared to attenuate over longer follow-up. Fifth, because serum albumin was not measured systematically during the study period, CAR could be calculated only in a subset of patients (*n* = 341 at admission and *n* = 286 at discharge), which reduced the statistical power of CAR-based Cox regression analyses. Although patients with and without available discharge CAR data had similar SOFA scores and 6-month mortality rates (*p* = 0.483 and *p* = 0.599, respectively), differences in APACHE II scores, ICU length of stay, and 28-day mortality indicated that non-random missingness and potential selection bias cannot be excluded. Despite these limitations, the study was conducted in a high-volume tertiary ICU with standardized management and follow-up protocols, supporting the internal validity of the findings. Future multicenter prospective studies are needed to confirm the generalizability of these results.

## 5. Conclusions

In conclusion, inflammatory biomarkers measured at ICU discharge, particularly NLR, remain predictive of early post-ICU mortality in patients with sepsis and septic shock, while their prognostic value appears to diminish over longer follow-up periods. An NLR value of ≥5 at ICU discharge may help identify patients at high risk for early mortality. These findings suggest that simple and routinely available biomarkers such as NLR can serve as practical tools for early post-ICU risk stratification and patient management. As this was a single-center study conducted in a chest diseases–specialized ICU with a high prevalence of COPD, these findings should be validated in non-specialty, mixed ICU populations before broader generalization.

## Figures and Tables

**Table 1:** Baseline demographic, clinical characteristics, and laboratory findings of patients with sepsis and septic shock.

	Group Sepsis (*n* = 291)	Group Septic Shock (*n* = 170)	*p* Value
Gender, male	179 (62)	106 (62)	0.86
Age, year	65 (54–74)	70 (63–79)	<0.001
Body mass index (kg/m^2^)	23 (21–28)	23 (20–30)	0.50
**Co-morbid diseases**			
COPD	164 (58)	111 (65)	0.07
Hypertension	121 (42)	92 (54)	0.010
Diabetes mellitus	63 (22)	46 (27)	0.19
Coronary artery diseases	34 (12)	35 (21)	0.010
**At the ICU admission**			
APACHE-II score	17 (15–22)	23 (19–29)	<0.001
SOFA score	3 (2–4)	5 (4–7)	0.001
**Mechanical ventilation**			
Invasive	75 (26)	137 (81)	<0.001
Non-invasive	205 (70)	128 (75)	0.26
Failure of noninvasive	23 (8)	61 (36)	<0.001
Length of ICU stay (days)	6 (3–8)	9 (7–14)	<0.001
**Inflammatory biomarkers**			
**At the ICU admission**			
CRP, mg/dL	78 (26–158)	87 (25–161)	0.99
NLR	9.77 (5.38–16.67)	12.07 (6.97–20.17)	0.024
PLT/MPV, ×10^3^	31 (22–42)	28 (19–38)	0.016
PLR	245 (145–392)	280 (160–488)	0.11
CAR	28.2 (7.8–59.1)	29.7 (10.0–67.5)	0.68
**Discharge from ICU**			
CRP, mg/dL	34 (16–75)	46 (23–85)	0.007
NLR	5.25 (3.68–8.02)	5.15 (3.37–8.16)	0.36
PLT/MPV, ×10^3^	28 (21–40)	25 (18–37)	0.010
PLR	200 (128–314)	195 (123–275)	0.27
CAR	14.7 (7.0–31.3)	21.3 (10.4–40.9)	0.005
**Mortality after ICU Discharge *n* (%)**			
28th day	29 (10)	40 (24)	<0.001
6th month	78 (27)	75 (44)	<0.001
One year	103 (35)	89 (52)	<0.001
Two year	165 (57)	120 (71)	0.003

Values are presented as median (IQR) or *n* (%). *p*-value < 0.05 is statistically significant. APACHE: Acute Physiologic and Chronic Health Evaluation; CAR: CRP-to-albumin ratio; COPD: chronic obstructive pulmonary diseases; CRP: C-reactive protein; ICU: intensive care unit; NLR: neutrophil-to-lymphocyte ratio; PLR: platelet-to-lymphocyte ratio; PLT/MPV: platelet/mean platelet volume; SOFA: Sepsis-related Organ Failure Assessment.

**Table 2:** Inflammatory biomarkers in survivor and non-survivor patients with/without septic shock at 28-day and 6-month follow-up.

	28th Day Mortality						6th Month Mortality					
Total	Admission			Discharge			Admission			Discharge		
	Survivor	Non-Survivor	*p*-Value	Survivor	Non-Survivor	*p*-Value	Survivor	Non-Survivor	*p*-Value	Survivor	Non-Survivor	*p*-Value
APACHE-II	19 (16–24)	22 (18–27)	<0.001	-	-	-	18 (15–23)	21 (17–27)	<0.001	-	-	-
SOFA score	3 (2–5)	5 (3–7)	0.002	-	-	-	3 (2–5)	4 (2–5)	0.07	-	-	-
NLR	9.9 (5.0–17.7)	14.4 (9.6–25.2)	<0.001	4.9 (3.4–7.5)	7.4 (4.5–11.1)	<0.001	9.9 (5.0–17.7)	12.2 (6.8–20.8)	0.037	5.0 (3.5–7.5)	5.8 (3.7–9.3)	0.024
PLR	240 (139–415)	322 (239–551)	0.001	196 (125–285)	202 (140–391)	0.14	235 (138–408)	296 (180–453)	0.012	192 (124–276)	211 (140–343)	0.06
PLT/MPV	30 (21–40)	31 (22–44)	0.56	27 (20–39)	26 (18–38)	0.25	29 (20–40)	30 (22–43)	0.25	27 (20–38)	27 (20–40)	0.86
CAR	25.0 (7.4–59.1)	44.3 (20.8–83.8)	0.004	16.5 (7.1–29.6)	30.9 (11.3–60.8)	<0.001	24.8 (7.6–58.1)	35.5 (10.4–78.8)	0.09	14.3 (6.8–25.3)	28.0 (11.2–50.5)	<0.001
CRP	73 (24–156)	119 (53–178)	0.010	37 (17–70)	73 (33–99)	<0.001	73 (25–156)	92 (33–170)	0.16	34 (17–64)	57 (24–97)	<0.001
**Group Septic Shock (** ***n* = 170)**												
APACHE-II	22 (19–28)	23 (20–30)	0.49	-	-	-	22 (19–26)	24 (20–30)	0.06	-	-	-
SOFA score	5 (4–7)	6 (4–9)	0.14	-	-	-	5 (4–7)	5 (4–7)	0.91	-	-	-
NLR	10.9 (5.2–18.9)	15.7 (9.3–25.2)	0.045	4.6 (2.9–7.1)	8.2 (4.8–11.0)	<0.001	10.7 (4.9–18.3)	14.4 (8.9–21.3)	0.10	4.7 (3.3–6.6)	5.9 (3.4–9.3)	0.040
PLR	255 (139–484)	322 (245–551)	0.044	195 (121–253)	199 (135–380)	0.21	242 (152–488)	298 (199–483)	0.15	184 (121–252)	198 (124–326)	0.29
PLT/MPV	28 (19–38)	27 (18–41)	0.94	26 (20–37)	21 (13–39)	0.16	29 (19–38)	27 (19–38)	0.47	27 (20–38)	23 (16–38)	0.053
CAR	23.6 (6.8–59.4)	42 (22–84)	0.021	17.7 (8.6–33.2)	30.2 (17.4–60.8)	0.005	21.9 (6.6–60.4)	36.2 (12.7–79.6)	0.12	17.1 (7.1–27.7)	29.3 (17.1–51.9)	<0.001
CRP	69 (21–145)	117 (48–176)	0.031	43 (20–79)	63 (46–92)	0.003	66 (17–159)	95 (40–175)	0.15	39 (18–63)	62 (41–94)	<0.001
**Group Sepsis (*n* = 291)**												
APACHE-II	17 (15–21)	21 (17–25)	0.015	-	-	-	17 (15–21)	19 (16–24)	0.014	-	-	-
SOFA score	3 (2–4)	3 (2–5)	0.29	-	-	-	3 (2–4)	3 (2–4)	0.78	-	-	-
NLR	9.3 (5.0–16.4)	12.7 (9.7–23.3)	0.006	5.1 (3.6–7.7)	7.1(5.3–13.7)	0.003	9.5 (5.0–16.5)	10.7 (6.0–19.6)	0.37	5.1 (3.6–7.6)	5.5 (3.9–8.2)	0.15
PLR	236 (139–390)	321 (211–563)	0.022	199 (128–309)	210 (154–399)	0.23	235 (135–390)	295 (177–422)	0.09	194 (125–302)	223 (144–376)	0.06
PLT/MPV	31 (22–42)	32 (28–48)	0.14	28 (21–40)	29 (25–36)	0.75	30 (21–41)	33 (28–47)	0.008	27 (20–38)	30 (24–41)	0.06
CAR	26 (7.6–58.6)	46 (20.3–81.3)	0.08	14 (6.5–26.0)	31 (10.4–59.4)	0.020	26 (8–57)	33 (8–78)	0.40	12 (6–25)	23 (9–47)	0.010
CRP	75 (26–156)	131 (58–179)	0.11	32 (15–66)	75 (22–116)	0.021	78 (27–156)	76 (26–165)	0.60	30 (16–64)	48 (17–98)	0.10

Values are presented as median (IQR) or *n* (%). *p*-value < 0.05 is statistically significant. APACHE: Acute Physiologic and Chronic Health Evaluation; CAR: CRP-to-albumin ratio; CRP: C-reactive protein; NLR: neutrophil-to-lymphocyte ratio; PLR: platelet-to-lymphocyte ratio; PLT/MPV: platelet/mean platelet volume; SOFA: Sepsis-related Organ Failure Assessment.

**Table 3:** The cut-off values of biomarkers with receiver operating characteristic curve Area Under the Curve values for follow-up mortality in sepsis patients (including with/without septic shock).

	Admission							Discharge						
Mortality		95% CI							95% CI					
28th Day	Area	Lower Bound	Upper Bound	Cut-Off	Sensitivity	Specificity	*p*-Value	Area	Lower Bound	Upper Bound	Cut-Off	Sensitivity	Specificity	*p*-Value
APACHE-II	0.63	0.57	0.70	20	63	58	<0.001	-	-	-	-	-	-	-
SOFA score	0.62	0.54	0.69	4	62	65	0.002	-	-	-	-	-	-	-
NLR	0.64	0.57	0.71	11.7	64	57	<0.001	0.67	0.60	0.74	5.0	67	55	<0.001
PLR	0.63	0.56	0.69	290	63	59	0.001	0.56	0.48	0.63	160	56	38	0.14
PLT/MPV	0.52	0.45	0.60	28	52	41	0.56	0.46	0.38	0.53	24	54	41	0.25
CAR	0.62	0.55	0.70	36	62	57	0.004	0.68	0.60	0.76	16.5	68	52	<0.001
CRP	0.61	0.53	0.68	96	61	55	0.010	0.66	0.59	0.73	44	66	57	<0.001
**6th month**														
APACHE-II	0.68	0.60	0.75	20.0	68	63	<0.001	-	-	-	-	-	-	-
SOFA score	0.60	0.52	0.68	4.0	60	65	0.016	-	-	-	-	-	-	-
NLR	0.58	0.50	0.66	11.3	58	64	0.06	0.59	0.51	0.67	5.0	59	59	0.026
PLR	0.58	0.50	0.66	265	58	66	0.06	0.52	0.44	0.61	180	52	50	0.59
PLT/MPV	0.49	0.41	0.58	30	51	47	0.89	0.43	0.35	0.51	27	57	50	0.93
CAR	0.62	0.54	0.69	36	62	53	0.005	0.69	0.61	0.76	13	69	53	<0.001
CRP	0.61	0.53	0.68	35	61	58	0.009	0.66	0.58	0.73	18	66	63	<0.001
**Long term (at least 2 year)**														
APACHE-II	0.66	0.58	0.73	18	66	58	<0.001	-	-	-	-	-	-	-
SOFA score	0.55	0.46	0.63	3.0	55	50	0.27	-	-	-	-	-	-	-
NLR	0.56	0.48	0.64	10.5	56	53	0.12	0.54	0.46	0.62	4.3	54	54	0.31
PLR	0.58	0.50	0.66	240	58	49	0.044	0.55	0.47	0.63	179	55	53	0.26
PLT/MPV	0.52	0.44	0.60	30	52	45	0.57	0.46	0.38	0.54	28.5	54	52	0.34
CAR	0.55	0.47	0.64	33	55	54	0.18	0.62	0.54	0.69	13	62	50	0.005
CRP	0.56	0.48	0.64	85	55	54	0.15	0.60	0.52	0.68	33.5	60	54	0.018

*p*-value < 0.05 is statistically significant. APACHE: Acute Physiologic and Chronic Health Evaluation; CAR: CRP-to-albumin ratio; CRP: C-reactive protein; NLR: neutrophil-to-lymphocyte ratio; PLR: platelet-to-lymphocyte ratio; PLT/MPV: platelet/mean platelet volume; SOFA: Sepsis-related Organ Failure Assessment.

**Table 4:** Area Under the Curve values for mortality to the biomarkers in septic shock patients.

Mortality	Admission							Discharge						
28th Day	Area	95% CI		Cut-Off	Sensitivity	Specificity	*p*-Value	Area	95% CI		Cut-Off	Sensitivity	Specificity	*p*-Value
		Lower	Upper						Lower	Upper				
APACHE-II	0.54	0.44	0.64	22.0	58	50	0.45	-	-	-	-	-	-	-
SOFA score	0.57	0.46	0.68	5.0	57	50	0.22	-	-	-	-	-	-	-
NLR	0.61	0.51	0.71	12.8	61	58	0.045	0.70	0.60	0.79	5.0	72	64	<0.001
PLR	0.61	0.51	0.70	285	62	58	0.044	0.57	0.45	0.68	160	64	37	0.26
PLT/MPV	0.50	0.38	0.61	27	51	47	0.94	0.43	0.31	0.54	24	54	56	0.21
**6th month**														
APACHE-II	0.60	0.51	0.68	22.5	60	56	0.033	-	-	-	-	-	-	-
SOFA score	0.50	0.41	0.59	5.0	50	45	0.95	-	-	-	-	-	-	-
NLR	0.58	0.49	0.66	11.8	59	64	0.10	0.60	0.51	0.69	5.0	58	53	0.031
PLR	0.56	0.48	0.65	285	56	59	0.15	0.54	0.45	0.64	170	55	45	0.32
PLT/MPV	0.47	0.38	0.56	27	57	47	0.47	0.41	0.33	0.50	25	60	43	0.056
**Long term (at least 2 year)**														
APACHE-II	0.61	0.53	0.70	21.0	62	50	0.019	-	-	-	-	-	-	-
SOFA score	0.49	0.40	0.59	5.5	48	52	0.88	-	-	-	-	-	-	-
NLR	0.59	0.49	0.68	10.0	65	50	0.08	0.54	0.45	0.64	4.5	61	50	0.38
PLR	0.63	0.54	0.72	250	64	62	0.010	0.53	0.44	0.62	180	54	52	0.53
PLT/MPV	0.50	0.41	0.60	27	53	46	0.96	0.46	0.37	0.55	29	63	60	0.42

*p*-value < 0.05 is statistically significant. APACHE: Acute Physiologic and Chronic Health Evaluation; NLR: neutrophil-to-lymphocyte ratio; PLR: platelet-to-lymphocyte ratio; PLT/MPV: platelet/mean platelet volume; SOFA: Sepsis-related Organ Failure Assessment.

**Table 5:** COX regression cut-off variables obtained from ROC analysis for mortality in septic patients discharged from the hospital.

Variables	Admission					Variables	Discharge				
	N	Hazard Ratio	95.0% CI for HR		*p*-Value		N	Hazard Ratio	95.0% CI for HR		*p*-Value
			Lower	Upper					Lower	Upper	
**28th day mortality predictor biomarkers**											
APACHE-II ≥ 20	461	1.75	0.88	3.48	0.11		-	-	-	-	-
SOFA score ≥ 4	461	1.30	0.67	2.51	0.44		-	-	-	-	-
NLR ≥ 11.7	451	1.22	0.58	2.57	0.6	NLR ≥ 5	454	2.44	1.24	4.80	0.010
PLR ≥ 290	451	1.91	0.90	4.05	0.09	PLR ≥ 160	454	0.61	0.31	1.22	0.16
PLT/MPV ≥ 28 × 10^3^	453	1.02	0.55	1.90	0.95	PLT/MPV ≥ 24 × 10^3^	455	1.19	0.62	2.30	0.6
CAR ≥ 36	341	3.26	1.09	9.76	0.035	CAR ≥ 16.5	286	1.80	1.00	3.23	0.051
CRP ≥ 96 mg/dL	362	0.55	0.18	1.64	0.28	CRP ≥ 44 mg/dL	400	1.65	0.86	3.15	0.13
**6th month follow-up mortality predictor biomarkers**											
APACHE-II ≥ 20	461	2.13	1.25	3.65	0.006		-	-	-	-	-
SOFA score ≥ 4	461	0.97	0.59	1.61	0.92		-	-	-	-	-
NLR ≥ 11.3	451	1.07	0.59	1.95	0.83	NLR ≥ 5	454	2.02	1.18	3.45	0.011
PLR ≥ 265	451	1.36	0.73	2.53	0.33	PLR ≥ 180	454	0.87	0.49	1.55	0.63
PLT/MPV ≥ 30 × 10^3^	453	0.90	0.56	1.46	0.67	PLT/MPV ≥ 27 × 10^3^	455	1.36	0.79	2.33	0.27
CAR ≥ 36	341	1.54	0.88	2.68	0.13	CAR ≥ 13	286	1.37	0.87	2.16	0.18
CRP ≥ 35 mg/dL	362	1.46	0.73	2.92	0.29	CRP ≥ 18 mg/dL	400	1.75	0.85	3.58	0.13
**2nd year mortality predictor biomarkers**											
APACHE-II ≥ 18	461	1.77	1.17	2.66	0.007		-	-	-	-	-
SOFA score ≥ 3	461	1.12	0.74	1.69	0.58		-	-	-	-	-
NLR ≥ 10.5	451	1.00	0.64	1.55	0.99	NLR ≥ 5	454	1.37	0.93	2.01	0.11
PLR ≥240	451	1.15	0.73	1.82	0.55	PLR ≥ 180	454	1.23	0.82	1.86	0.32
PLT/MPV ≥ 30 × 10^3^	453	1.08	0.74	1.56	0.70	PLT/MPV ≥ 28.5 × 10^3^	455	1.23	0.83	1.83	0.30
CAR ≥ 33	341	1.48	0.64	3.45	0.36	CAR ≥ 13	286	1.28	0.90	1.82	0.18
CRP ≥ 85 mg/dL	362	0.86	0.36	2.02	0.72	CRP ≥ 33.5 mg/dL	400	1.32	0.91	1.92	0.15

*p*-value < 0.05 is statistically significant. APACHE: Acute Physiologic and Chronic Health Evaluation; CAR: CRP-to-albumin ratio; CRP: C-reactive protein; NLR: neutrophil-to-lymphocyte ratio; PLR: platelet-to-lymphocyte ratio; PLT/MPV: platelet/mean platelet volume; SOFA: Sepsis-related Organ Failure Assessment.

## Data Availability

The datasets used and/or analyzed during the current study are available from the corresponding author on reasonable request.
